# Profiling High-Abundance Serum Proteins in the Corona of Nanodiamonds Using Mass Spectrometry

**DOI:** 10.1021/acs.langmuir.5c06674

**Published:** 2026-03-16

**Authors:** Mhikee Janella N. Descanzo, Yu-Chung Chen, Ming-Chi Chung, Nghiem Bich Ngoc Nguyen, Avinash A. Patil, Po-Chi Soo, Yu-Tze Horng, Chia-Liang Cheng, Huan-Cheng Chang, Ruey-Yi Chang, Wen-Ping Peng

**Affiliations:** † Department of Physics, 63373 National Dong Hwa University, Shoufeng, Hualien 97401, Taiwan; ‡ Department of Biochemical and Molecular Medical Sciences, 63373 National Dong Hwa University, Hualien 97401, Taiwan; § Institute of Atomic and Molecular Science, 38017 Academia Sinica, Taipei 10617, Taiwan; ∥ Department of Laboratory Medicine and Biotechnology, 59216 Tzu Chi University, Hualien 97004, Taiwan

## Abstract

Nanodiamonds (NDs) are promising agents for various biomedical applications. Upon entering the body, NDs interact with proteins to form a “protein corona” (PC), which influences their biological fate and cytotoxicity. In this study, 100 nm NDs were incubated with human serum, and MALDI-TOF MS was used to identify the major proteins dominating the corona on oxidized detonation ND (oxDND) and high-pressure high-temperature ND (HPHT ND). The results show that PC formation depends on serum protein abundance, protein affinity, kinetics, and ND properties. At a serum protein concentration of 5.3 μg/mL, both NDs are predominantly coated with apolipoprotein A1 (APO A1; Mw = 28,000 Da). As serum protein concentration decreased to 1.6 μg/mL, oxDND becomes dominated by human serum albumin (HSA; Mw = 66,500 Da), while HPHT ND exhibits a mixed APO A1-HSA corona. Differences in pore structure also influence protein binding: the larger pores of oxDND (9.41 nm) allow access to larger proteins like HSA, whereas smaller HPHT ND pores (3.39 nm) favor low molecular weight (Mw) proteins. Hemolysis assays indicated good hemocompatibility for both NDs, while cell viability assays revealed higher cytotoxicity for HPHT ND with a mixed corona compared to oxDND with an HSA-dominant corona. These findings highlight the importance of profiling high-abundance proteins in ND coronas and demonstrate how protein binding preferences modulate ND cytotoxicity.

## Graphical abstract

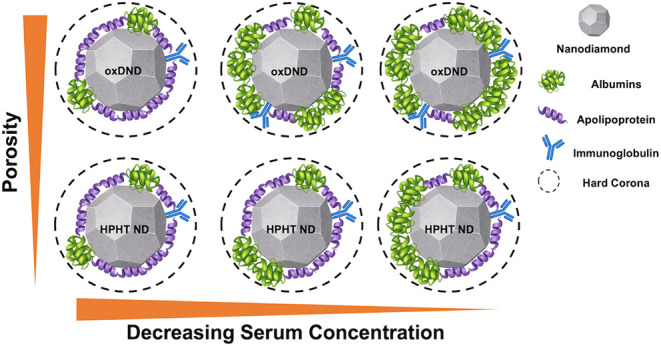

## 1 Introduction

Due to their physicochemical properties—including biocompatibility, easily modifiable surface, and optical stability—nanodiamonds (NDs) have been increasingly investigated in a range of biomedical applications such as drug delivery, cellular imaging, and therapeutics.^1,2^ Once exposed to the body, either by injection or inhalation, NDs are expected to interact with bodily fluids, such as blood. These bodily fluids contain proteins that can strongly and rapidly attach to the surfaces of NDs, eventually forming a so-called “protein corona” (PC). The PC is a complex and dynamic layer that affects the biological identity of nanoparticles (NPs), altering their cellular uptake, distribution, and potential toxicity.^3–5^ For instance, Ge et al. suggested that carbon nanotubes (CNTs) coated with proteins, such as fibrinogen, bovine serum albumin (BSA), and transferrin, showed less toxicity compared to their uncoated counterparts.^6^ The presence of these proteins prevents CNTs from direct cellular contact, leading to a decreased toxicity. Similarly, graphene oxide (GO) demonstrates dependence on PC formation in determining its cytotoxicity. Hu et al. showed that at low-serum conditions (1% fetal bovine serum, FBS), GO induces significant cellular damage.^7^ In contrast, high-serum environments (10% FBS) facilitate the formation of a protective PC, preventing direct membrane interactions and significantly lowering cytotoxic effects. However, the influence of the PC is not always protective. Barbalinardo et al. showed the apolipoprotein-enriched coronas increased the cellular uptake of silver NPs, leading to higher cytotoxicity. Thus, depending on its composition, the PC can either mitigate or promote NP toxicity.^8^ Since the composition of corona strongly affects the biological fate and cytotoxicity of NPs, understanding the factors that affect its evolution is an essential step that could lead to safer and more effective NP-based drug delivery and nanomedicine applications.^3^

The process of PC formation is influenced by multiple factors. The physical and chemical properties of NPs, such as size, shape, surface charge, surface functional groups, and porosity, are some of the important parameters that can be considered in the investigation of PC formation.^3,9,10^ For example, Schäffler et al. reported that smaller gold NPs (AuNPs) with a higher curvature bind significantly more proteins of all sizes as compared to larger AuNPs with a lesser curvature.^9^ Additionally, the biological conditions also play an important role in the evolution of PC.^11–14^ Partikel et al. compared the protein identity of coronas on poly­(dl-lactide-*co*-glycolide) (PLGA) NPs incubated in FBS and human serum.^13^ A higher number of immune response-related proteins was detected after the incubation of the NP with human serum. Furthermore, the PC composition dramatically changes when the human serum concentration varied, while it remained stable for FBS. Similarly, Lin et al. showed that serum concentration influences protein adsorption on mesoporous NPs.^14^ The results showed that increasing the serum concentration led to a dramatic increase in the number of smaller proteins (<25,000 Da). Although extensive research has been conducted on PC formation in different NPs, studies specifically addressing NDs remain limited.

The limited ND-focused literature offers valuable insights but also shows that important questions remain. For instance, Khanal et al. investigated corona formation on ∼5 nm pristine and aminated NDs using standard protein samples (BSA and fibronectin).^15^ In addition to this, they showed that reactive oxygen species (ROS) production and macrophage toxicity differ significantly when NDs interact with cells in the presence or absence of FBS in culture media. Garcia-Bennett et al. further explored how the surface chemistry of ∼1–50 nm NDs influences corona formation in FBS.^16^ Despite variations in surface functional groups, the top 30 adsorbed proteins were broadly similar. Consistent with other studies, preformed coronas reduced ROS generation in macrophages, especially on negatively charged NDs. Machová et al. examined ultrasmall (∼1–5 nm) hydrogenated versus oxidized detonation NDs (DNDs) and demonstrated that surface termination strongly affects selective protein adsorption and subsequent cellular interactions.^17^ These studies highlight the importance of ND size, surface chemistry, and the biological environment in the formation of PC. However, these existing reports focus primarily on small or surface-modified NDs and rely on nonhuman media such as FBS or purified protein systems. As a result, it remains unclear how larger NDs (∼100 nm) interact with high-abundance proteins in human serum. Addressing this gap is essential for understanding ND behavior in different biological environments and for guiding their safe and effective use in biomedical applications.

In the present study, the dominant proteins composing the PC on NDs were profiled using matrix-assisted laser desorption/ionization time-of-flight mass spectrometry (MALDI-TOF MS). Two commonly used types of diamond NPs, oxidized DND (oxDND) and high-pressure high-temperature ND (HPHT ND), were incubated with human serum to investigate how their distinct physicochemical properties^10^ determine the primary proteins forming their corona. Moreover, the study examined the impact of biological conditions on corona composition by varying human serum concentrations. Lastly, the cytotoxic effects of NDs toward red blood cells (RBC) and A549 cells were analyzed in relation to their corona profile, providing insights into how variations in surface-bound proteins influence cellular interactions and potential toxicity.

## 2 Materials and Methods

### 2.1 Materials and Chemicals

The commercially available acid-oxidized/purified HPHT ND with a size ∼100 nm was obtained from Kay Industrial Diamond (Boca Raton, FL, USA). The HPHT ND was used without additional treatment. The DND powder with a size of 200 nm was obtained from Taiwan Union Abrasives Corporation (Hsinchu County, Zhubei City, Taiwan). According to the manufacturer, the 200 nm ND powders were produced via a detonation method, followed by sieving to separate particles by size. However, this approach may introduce impurities into the resulting material. To address this, an additional air oxidation treatment^10^ was performed to obtain the final product: ∼100 nm oxDND. The human-male serum sample (SI–H4522) was from Sigma (St. Louis, Missouri, USA). According to the certificate of analysis provided by the manufacturer, the serum sample has a total protein concentration of 53 mg/mL. Sinapic acid (SA; 85429), α-cyano-4-hydroxy-cinnamic acid (CHCA; C2020), iodoacetamide (IAA; 144489), trifluoroacetic acid (TFA; L06374), and the sequencing-modified trypsin (T9201) were also purchased from Sigma. β-mercaptoethanol (β-ME; A15890–30) was from Thermo Fisher (Massachusetts, USA). Acetonitrile (ACN; 02002180) was purchased from Fitzgerald (Concord, MA, USA). The materials used for cell culture, such as Dulbecco’s Modified Eagle Medium (DMEM; 12800017), F12 (A5209401), and fetal bovine serum (FBS; 21700018), were purchased from Gibco (USA). Deionized (DI) water was purified using Milli-Q water purification system (Millipore, Billerica, MA).

### 2.2 Dynamic Light Scattering (DLS)

The hydrodynamic size distribution and zeta potential of oxDND and HPHT ND were measured using a Malvern Zetasizer Nano-ZS (Malvern Instruments Inc., United Kingdom) equipped with a 4 mW He–Ne laser operating at 632.8 nm. Measurements were conducted at 25 °C using a scattering angle of 173°. The viscosity and refractive index of the water (0.8872 cP and 1.330, respectively) were used for data analysis. Hydrodynamic diameters were reported based on z-average. Zeta potential values were determined using the Smoluchowski model. Each reported value represents the mean ± standard deviation (SD) of six independent measurements.

### 2.3 Brunauer–Emmett–Teller (BET) Isotherm Analysis

Before analysis, the ND samples were degassed overnight in a vacuum at 150 °C. Forty-point isotherms with nitrogen were collected over a relative pressure range of *p*/*p*_0_ = 0.05 – 1 (where *p*_0_ is the saturation pressure, 760 Torr for N_2_) using a Quantachrome surface area and porosity analyzer (USA). The BET isotherm was used to determine the specific surface area (SSA) of the samples. Meanwhile, the Barrett–Joyner–Halenda (BJH) method was used to estimate the pore volume and pore area distributions.

### 2.4 Serum Protein Adsorption on the Surface of NDs

Prior to protein extraction from serum samples, the ND powders were separately suspended in DI water at a concentration of 1 mg/mL and sonicated using ultrasound (Delta Ultrasonic Cleaner DC400H, New Taipei City, Taiwan) for 30 min. Then, a 10 μg/mL suspension of NDs was incubated with human serum at the desired total protein concentration (1.1–5.3 μg/mL; diluted in DI water) under constant movement on a vortex shaker. Afterward, the protein-loaded NDs were isolated by centrifugation in a microcentrifuge (Scilogex D3024 Microcentrifuge, CT, USA) at 16,060 g for 40 min. The supernatants were collected and stored for subsequent extraction experiments. The pellets were then washed with 500 μL of DI water to remove any unbound or excess proteins. Although the wash step is expected to remove any unbound proteins, an additional time-dependent experiment (Figure S1) was conducted to further assess protein attachment stability and to standardize the protocol used in this study. Based on the results, a 30 min incubation was selected for all subsequent experiments. The overall protocol used in this study is illustrated in Scheme 1.

**Scheme 1:**
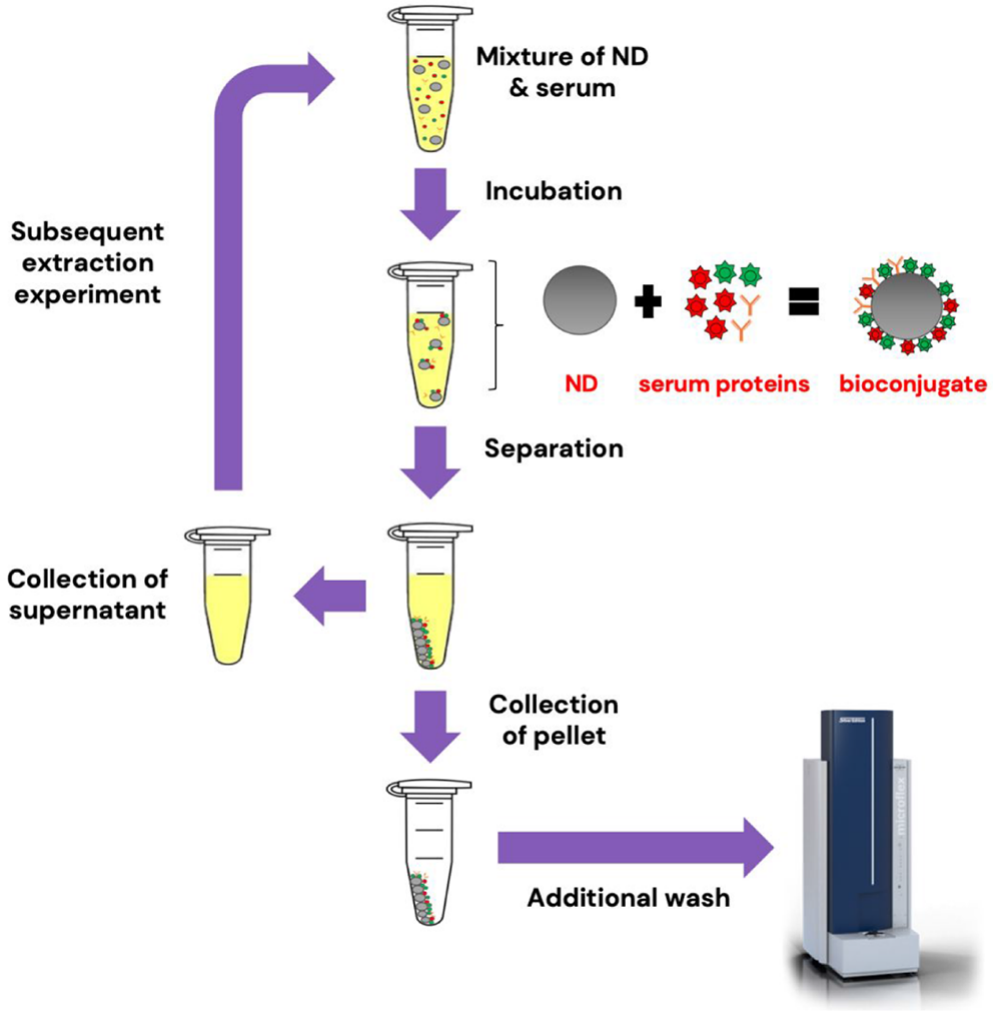
Adsorption of Proteins on the Surfaces of oxDND and HPHT ND[Fn sch1-fn1]

i In this study, the term “extraction” refers to the adsorption of different proteins onto the surfaces of NDs. “1st extraction” and “2nd extraction” denote the first and second adsorption experiments, respectively.

For mass spectrometric (MS) analysis, a solution of SA matrix (1 μL, 2 mg/100 μL prepared in 50% ACN/DI water with 0.1% TFA) was mixed with the pellet, and the mixture was deposited onto the MALDI target plate (polished stainless steel: MSP 96 Target Plate). The forced dried droplet (FDD) method was used to prepare the sample, ensuring homogeneous microcrystals prior to MS analysis.^10,18^

For size and zeta potential measurements after protein adsorption, the pellet was resuspended in 1 mL of DI water. Subsequently, 700 μL of the solution was transferred into a folded capillary tube, and the measurements were performed as described above.

### 2.5 On-Diamond Digestion

The on-diamond digestion method previously described by Soo et al.^19^ was followed to digest the proteins attached to the ND surfaces. To ensure the sufficient abundance of the target protein (*m*/*z* peak at 28,000), 5.3 μg/mL of serum sample was incubated with 10 μg/mL of oxDND. The protein-loaded ND pellet was then resuspended in a reducing buffer containing 2% β-ME (HOCH_2_CH_2_SH) and 25 mM ammonium bicarbonate (NH_4_HCO_3_). The mixture was incubated in a dark place for 10 min at room temperature. An alkylation buffer containing 380 mM IAA and 25 mM NH_4_HCO_3_ was then added, and the mixture was kept in the dark for another 10 min at room temperature. The mixture was centrifuged for 10 min at 21,380 g, and the supernatant was carefully removed. The resulting pellet was washed twice with DI water and once with 100% ACN. Finally, a trypsin solution containing 4.2 μM sequencing-modified trypsin and 25 mM NH_4_HCO_3_ was added to the pellet. The digestion was carried out for 16 h at 37 °C.

### 2.6 MALDI-TOF MS Analysis

Mass spectra were acquired using a Microflex MALDI-TOF mass spectrometer (Bruker Daltonics, Bremen, Germany) equipped with a 337 nm nitrogen laser. For direct protein analysis, the protein-loaded NDs were analyzed in linear positive mode. The microchannel plate detector was set to 1742 V, and the voltages for ion source 1, ion source 2, and the einzel lens were 18.20, 13.10, and 9.10 kV, respectively. Spectra were acquired over an *m*/*z* range of 5,000–250,000 with approximately 400 laser shots accumulated per sample and processed using FlexAnalysis software (Bruker Daltonics).

For the analysis of digested samples, measurements were performed in reflectron positive mode to enhance mass accuracy and resolution for peptide detection. A CHCA matrix solution (prepared as described above; 2 mg/100 μL) was used, and samples were spotted on a stainless-steel MALDI target plate and air-dried. The microchannel plate detector was set to 1,684 V, and voltages for ion source 1, ion source 2, einzel lens, and the reflector were 19.00, 14.50, 9.80, and 20.00 kV, respectively. Mass spectra were acquired in the *m*/*z* range of 500–5,000 with 200 laser shots and externally calibrated using a myoglobin-cytochrome c protein mixture.

### 2.7 Cell Culture

The A549 cell line, derived from human lung adenocarcinoma, was kindly provided by Dr. R. Y. Chang from the Department of Biochemical and Molecular Medical Sciences, National Dong Hwa University. The cells were cultured in DMEM/F-12 medium (Gibco, USA) supplemented with 10% FBS, 1% penicillin–streptomycin, and 1% l-glutamine, and maintained at 37 °C in an incubator containing 5% CO_2_.

### 2.8 Evaluation of the Cytotoxic Activity of NDs with and without PC

#### 2.8.1 Hemolysis of Erythrocytes by ND

Hemolysis was assessed to evaluate the effect of the PC on the cytotoxicity of the two NDs. Following previously described hemolysis assay methods,^20,21^ commercially available human blood (Formosa Biomedical Technology Corp., Taipei, Taiwan) was prepared at a final concentration of 6% in saline solution (0.9% sodium chloride, NaCl). NDs were dispersed in saline solution and added to the blood at a final concentration of 100 μg/mL. A volume of 500 μL of the blood-ND mixture was incubated at 37 °C for 1 h, followed by centrifugation at 1,000 g for 5 min. After centrifugation, 200 μL of the supernatant from each sample was transferred to a 96-well microplate, and absorbance was measured at 540 nm using a microplate reader. Saline solution served as the negative control, while 0.1% (v/v) Triton X-100 was used as the positive control to induce complete hemolysis. The hemolysis ratio (%) was calculated using the following formula:

$$\mathrm{hemolysis ratio}(\%)=\left(\frac{OD_{\mathrm{sample}}-OD_{\mathrm{negative control}}}{OD_{\mathrm{positive control}}-OD_{\mathrm{negative control}}}\right)\times 100$$

#### 2.8.2 The Effects of ND on Cell Morphology

To observe the morphology of cells exposed to ND using a microscope, A549 cells were seeded at a density of 3 × 10^4^ cells per well on 12-well plates. The cells were allowed to adhere to the coverslip placed inside each well overnight (37 °C, 5% CO_2_). For the *without corona* condition, the medium was replaced with serum-free medium (SFM) containing 100 μg/mL NDs. For the *with preformed corona* condition, 100 μg/mL NDs were preincubated with 16 μg/mL total serum proteins—comparable to the 10 μg/mL ND: 1.6 μg/mL protein ratio as shown in Figure S2. The ND-protein complexes were then dispersed in SFM and added to the cells. After 4 h of incubation, the ND-containing medium was removed and replaced with fresh SFM. The cells were allowed to grow for an additional 12 h. The samples were then washed three times with PBS and fixed with 10% formaldehyde solution for 15 min. Images were captured using a Leica TCS SP8 microscope (Mannheim, Germany).

#### 2.8.3 The Effects of ND on Cell Viability

The viability of A549 cells exposed to NDs, with and without PC, was determined using the CCK-8 assay (Elabscience, Japan). Following the manufacturer’s protocol, A549 cells were seeded at a density of 5 × 10^3^ cells per well in 96-well plates and allowed to attach overnight. Similar to the method described above, the cells were treated with 100 μg/mL NDs dispersed in SFM without corona condition. For the with preformed corona condition, 100 μg/mL NDs were preincubated with 16 μg/mL total serum proteins, then dispersed in SFM and applied to cells. The cell-ND mixtures were incubated for 4 h. After incubation, the cells were washed three times with PBS, and 100 μL of fresh SFM was added to each well. Finally, 10 μL of CCK-8 buffer was added to each well, and the optical density (OD) was measured at 450 nm using a microplate reader.

## 3 Results and Discussion

### 3.1 Characterization of NDs

Key factors such as the size, shape, surface charge, surface functional groups, and porosity play a significant role in the adsorption of proteins on NPs. In this study, DLS, FTIR, and BET methods were used to characterize both oxDND and HPHT ND.

As shown in Table 1, oxDND and HPHT ND exhibit comparable hydrodynamic sizes of 144 ± 2 nm and 158 ± 2 nm, respectively. Both ND systems have low polydispersity indices (PDIs), indicating their monodispersity in aqueous solutions. Additionally, both oxDND and HPHT ND display similar zeta potentials of −49 mV. The surface functional groups present on the surface of the two NDs were verified using FTIR spectroscopy. Figure S3 shows the spectra obtained from the measurements. Consistent with previous studies, the FTIR spectra reveal distinct features in the 3200–3600, 1730–1820, and 1200–1500 cm^–1^ regions. These observed bands are attributed to O–H from physically adsorbed water, C=O stretching,^10,22^ and several complex characteristics of ND surfaces (C–C, C–OH, COO^–^, etc.),^23–25^ respectively. The results from the size, zeta potential, and FTIR measurements indicate significant similarities between oxDND and HPHT ND. These results help the study narrow-down the key factors that may influence the formation of the PC on the ND surfaces.

**Table 1:** Hydrodynamic Diameter, Polydispersity Index (PdI), and Zeta Potential of oxDND and HPHT ND Measured Using DLS^a^

Sample	Hydrodynamic Diameter (nm)	PdI	ζ-Potential (mV)
oxDND	144 ± 2	0.20 ± 0.02	-49 ± 3
HPHT ND	158 ± 2	0.13 ± 0.02	-49 ± 0.9

a Data are presented as mean ± standard deviation (*n* = 6).

Meanwhile, BET isotherm analysis (Table 2) revealed that oxDND has a larger SSA of 268.4 m^2^/g, compared to HPHT ND, which has an SSA of 34.7 m^2^/g. Additionally, the two types of NDs exhibit significant differences in cumulative pore volume and average pore diameter. The estimated pore volume and pore diameter for oxDND are 2.65 cc/g and 9.41 nm, respectively, while HPHT ND has a pore volume of 0.367 cc/g and a pore diameter of 3.39 nm. It is important to note that these pore characteristics mainly reflect the spaces formed between ND aggregates in dry powders rather than intrinsic porosity of the individual ND particle. Previous Raman and X-ray photoelectron spectroscopy (XPS) analyses from our group have also shown that oxDND possesses a more oxidized and sp^2^-rich surface compared to less oxidized, sp^3^-dominant HPHT ND (summarized in Section 1 of the Supporting Information). Combined with the BET/BJH results, these established differences further support examining how each ND type develops a distinct PC.

**Table 2:** Specific Surface Area (SSA), Cumulative Desorption Volume, and Desorption Diameter of oxDND and HPHT ND

	Multipoint BET	BJH Analysis	
Sample	SSA (m^2^/g)	Pore Volume (cc/g)	Pore Diameter (nm)
oxDND	268.4	2.65	9.41
HPHT ND	34.7	0.37	3.39

### 3.2 MALDI-TOF MS Analysis of High-Abundance Serum Proteins in the PC of NDs

MALDI-TOF MS was used to identify the major proteins on oxDND and HPHT ND, providing insight into how ND type and serum conditions affect protein binding.

Before examining corona composition, consecutive ND-protein extractions were performed to assess the maximum protein adsorption per ND and to verify that the serum concentrations used were sufficiently high relative to the NP surface sites. At a total serum protein concentration of 5.3 μg/mL, three extractions were possible for both oxDND and HPHT ND (Figures S4a, S5a). As the concentration decreased to 2.7 μg/mL (Figures S4b, S5b) and 1.6 μg/mL (Figures S4c, S5c), the number of extractions dropped to two. The signal intensity in the second extraction at 1.6 μg/mL is noticeably lower than that of the 2.7 μg/mL, suggesting fewer proteins remained in the supernatant. At 1.1 μg/mL (Figures S4d, S5d), a single extraction was sufficient, indicating near-complete adsorption of available proteins. These results indicate that both ND types adsorb approximately 1.1–1.3 μg/mL of serum proteins per extraction. Despite the larger SSA of oxDND (268.4 m^2^/g) compared to HPHT ND (34.7 m^2^/g), MS analysis showed that both ND types adsorbed nearly the same total amount of serum proteins. The Bradford assay, a widely used quantitative colorimetric technique, was also applied to assess total protein adsorption. At 2.7–5.3 μg/mL serum protein, the Bradford assay overestimated the amount of adsorbed protein compared to MALDI-TOF MS (Figure S6b, S6c), consistent with known limitations of this assay.^10^ Additionally, due to the assay’s detection limit, it was not possible to measure adsorption at the lowest serum concentrations used in MALDI-TOF MS. Nonetheless, these combined results confirm that the serum concentrations explored here are sufficient to approach ND surface saturation, validating subsequent MALDI-TOF MS analysis.

With this validation, MALDI-TOF MS analysis was used to identify the high-abundance serum proteins on the ND surfaces. The MS results show an interesting difference between the proteins attached to the two NDs as the human serum concentration varied. At a total serum protein concentration of 5.3 μg/mL (Figure S4a), the oxDND was dominated by proteins with lower *m*/*z* ranging from 9,000 to 28,000 (Figure 1a and Figure S4). A significant change in the protein identity occurred as the protein concentration decreased to 1.6 μg/mL (Figure S4c), with a dominant protein peak at 66,500. Meanwhile, the lower *m*/*z* observed at 9,000 completely vanished, while the peaks at 14,000 and 28,000 decreased significantly. As the concentration of the human serum protein was further decreased to 1.1 μg/mL (Figure S4d), the protein peak at 66,500 continued to dominate the surface of oxDND. A different scenario was observed with the MS analysis of PC on HPHT ND (Figure 1b and Figure S5). At total human serum protein concentration of 5.3 μg/mL (Figure S5a), the mass spectrum was still dominated by lower mass proteins. As the protein concentration was decreased to 1.6 μg/mL (Figure S5c), the protein peak at 66,500 increased. Interestingly, even though there was an increase in higher mass protein, the intensity of the lower mass proteins remained to be stable. The intensities of both low and high mass protein peaks remained to be relatively stable as the serum concentration was further decreased to 1.1 μg/mL. The bottom-up approach was used to identify the proteins on the surface of the two NDs. Using this approach, the adsorbed proteins were tryptically digested, and the resulting peptides were identified using the Mascot search engine. Figure 2 shows the mass spectra obtained from the three replicates of the trypsin digestion experiment. The complete list of matched peptides from the Mascot search engine is provided in Table S2. The results revealed that the peak observed at 28,000 corresponds to apolipoprotein A1 (APO A1), with a significant Mascot score of 86 and a protein sequence coverage of 71%. Additionally, the peak at 66,500 is identified to be the human serum albumin (HSA), with a Mascot score of 58 and a protein sequence coverage of 54%. Taken together, these results indicate that at a higher serum protein concentration (5.3 μg/mL), both oxDND and HPHT ND were predominantly coated with APO A1. As the total serum concentration decreased to 1.6 μg/mL, oxDND exhibited an HSA-dominant corona, whereas HPHT ND display a mixed APO A1-HSA corona. Upon further reduction in protein concentration, HSA remained dominant on oxDND, while both APO A1 and HSA persisted on HPHT ND. In addition to these abundant proteins, a distinct immunoglobulin G (IgG) peak was consistently detected across all serum protein concentrations, though its intensity remained relatively stable and did not show the concentration-dependent changes observed for HSA and APO A1. It should be noted that these observations were obtained under MALDI-TOF MS-compatible conditions, with experiments conducted in DI water rather than physiologically relevant media such PBS due to salt limitations. Nonetheless, the observed trends are consistent with prior studies, supporting the relevance of these findings. The results align with the general Vroman model,^26–28^ wherein initially adsorbed proteins can be replaced by higher-affinity proteins as the environmental conditions change. Similar behavior has been reported when NPs are incubated in 0–100% serum, showing shifts in protein patterns and additional appearance of low molecular weight proteins at higher serum concentrations.^29^ These observations are further consistent with previous studies,^13,14,30–32^ and with the framework described by Partikel et al.,^13^ which proposes that highly abundant, lower-affinity proteins dominate NP surfaces at low serum concentrations, while lower-abundance proteins with higher affinity increasingly compete for binding and displace them as serum concentration increases.

**Figure 1:**
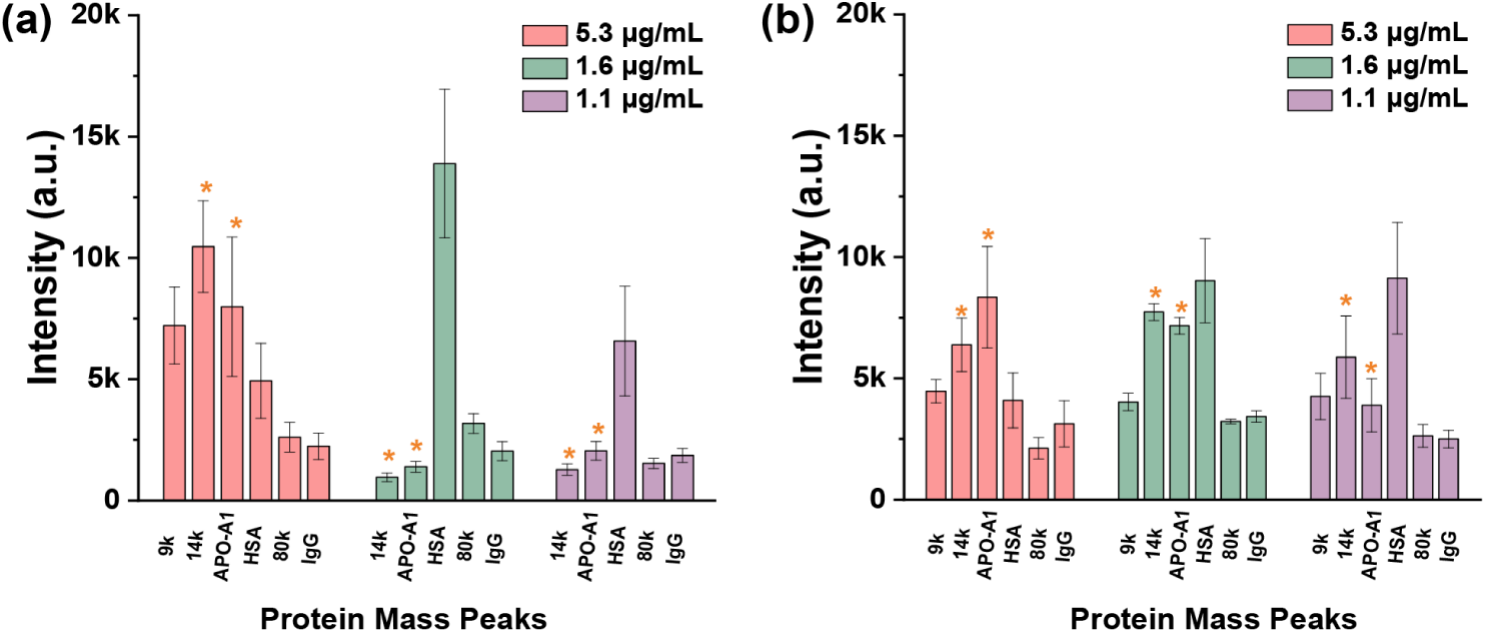
Dominant peaks observed in MALDI-TOF MS analyses of ND-human serum protein complexes were replotted into a bar graph to show the effects of both the serum concentration and the physicochemical properties of NDs on the protein corona composition; (a) oxDND and (b) HPHT ND. Data are presented as mean ± standard deviation (*n* = 6).

**Figure 2:**
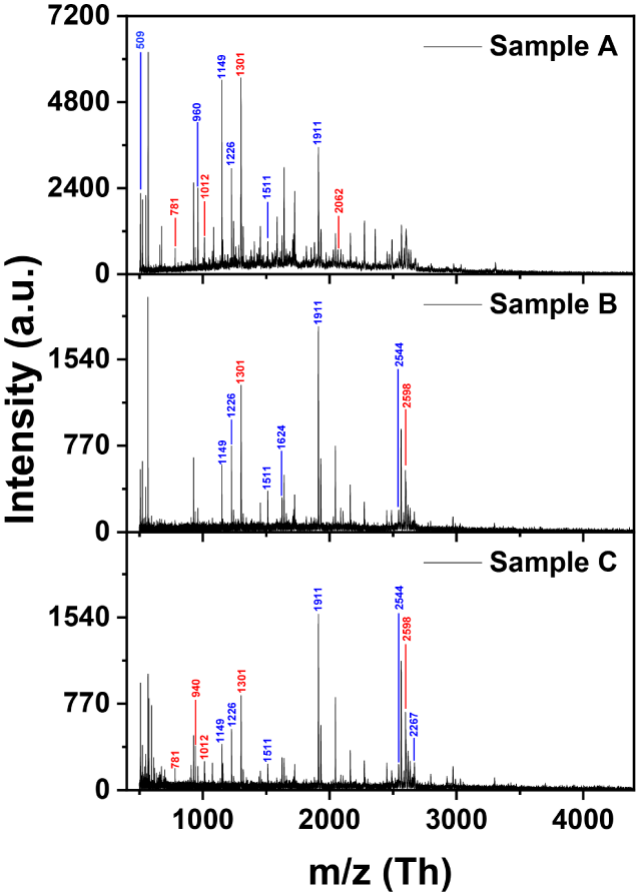
Tryptic peptide mass spectra of serum proteins adsorbed on oxDND. Triplicate analyses confirmed the consistent identification of HSA and APO A1. Peaks labeled in blue (*m*/*z* values) correspond to HSA, while those in red correspond to APO A1.

Another factor that may contribute to the competition between these proteins is the primary differences between oxDND and HPHT ND, namely their cumulative pore characteristics and SSA. Although BET measurements reflect properties in dry state rather than in aqueous solution, they still provide a useful indication of relative porosity. At fixed serum protein concentrations, a noticeable difference between the corona composition of oxDND and HPHT ND was observed. At 1.6 and 1.1 μg/mL of serum proteins, the PC of oxDND with an average *d*_pore_ = 9.41 nm is dominated by HSA, which has a considerably larger Mw of 66,500 Da. Contrastingly, HPHT ND with an average *d*_pore_ = 3.39 nm contains a corona with a seemingly competitive amount of low Mw proteins (APO A1). These observations support an earlier model showing that NPs with pore diameters below 7.4 nm can discriminate based on protein size.^33^ Smaller pores restrict higher Mw proteins from accessing the entrance by trapping low molecular proteins inside, while larger pores allow greater access for high Mw proteins to the internal porosity. Importantly, pore structure and SSA represent only part of the explanation, with surface chemistry differences previously observed by Raman and XPS (Section 1 of the Supporting Information) also likely contributing to selective corona formation.

### 3.3 Effects of PC Formation on the Size and Zeta Potential of NDs

The adsorption of different proteins onto NP surfaces affects their properties, therefore influencing their biological fate. Hence, changes in ND hydrodynamic size and zeta potential were tracked at varying serum concentrations. These measurements served as a complementary support to MALDI-TOF MS, which identified the high-abundance proteins adsorbed on each ND.

Figure 3 and Table S1 summarize the measured hydrodynamic size and zeta potential of the two NDs after exposure to human serum. In general, an increase in the size of NP is expected upon protein adsorption. The size of oxDND significantly increased to 239 nm after its exposure to a higher serum protein concentration (5.3 μg/mL). As the oxDND was exposed to lower concentrations of serum proteins, the increase in size became significantly smaller too. Notably, there is a substantial size reduction as the serum protein concentration decreased from 5.3 μg/mL to 1.6 μg/mL. This pattern is consistent with MS results and could indicate a significant change in the dominant proteins adsorbed on the oxDND surface. Meanwhile, HPHT ND exhibited a similar overall pattern, with an increase in size at higher serum protein concentrations and a decrease in size at lower concentrations. However, the decrease in size of HPHT ND observed at lower serum concentration (1.6 μg/mL) was less dramatic, further suggesting that the PC composition remained relatively stable on its surface.

**Figure 3:**
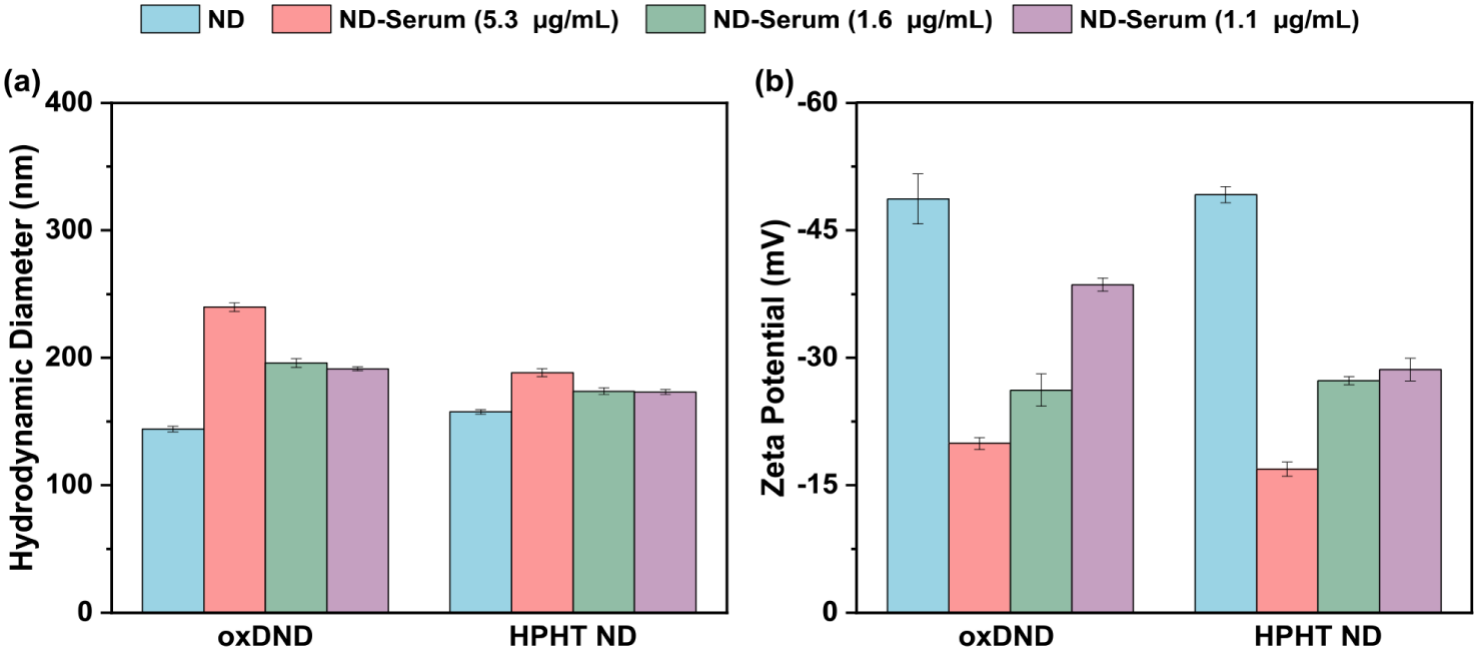
(a) Hydrodynamic diameter and (b) zeta potential of oxDND and HPHT ND before and after the incubation with serum at different concentrations: 5.3, 1.6, and 1.1 μg/mL. Data are presented as mean ± standard deviation (*n* = 6).

The adsorption of proteins onto negatively charged NPs typically results in a lower zeta potential, depending on the type and number of proteins present.^9,13,30^ After incubating oxDND with a serum protein concentration of 5.3 μg/mL, its measured zeta potential drastically decreased to −20 mV. As the serum protein concentration decreased, the zeta potential increased. Similar to the size measurements, a significant change in the zeta potential of oxDND was observed when the serum protein concentration was reduced to 1.6 μg/mL. This further verifies a shift in the PC evolution on the oxDND surface with decreasing serum protein concentration. A similar observation was made for HPHT ND, where the measured zeta potential increased as the serum protein concentration decreased. A significant rise in zeta potential from −49 mV to −17 mV was seen when the serum protein concentration dropped from 5.3 μg/mL to 1.6 μg/mL. This could indicate a change in the PC composition; however, it is not fully consistent with both the MS and size measurement results. A possible explanation is that the substantial change in zeta potential primarily reflects differences in surface coverage rather than the specific identity of adsorbed proteins themselves. As the total protein concentration decreased, the HPHT ND surface became less densely coated, causing the measured zeta potential to approach that of the bare, negatively charged HPHT ND.

### 3.4 Varying PC Composition on the Cytotoxicity of NDs

Both mass spectrometry and DLS measurements have shown that the formation of PC on the surface of NDs alters their surface properties. These changes confer a new biological identity to the NDs, which could influence not only their biological fate but also their toxicity. Evaluating how different PC compositions affect cell viability and morphology is critical in the NP field, as it informs the safe design of NPs and predicts their biological behavior.^4,34^ Herein, hemolysis assay, microscopy, and CCK-8 assay were used to briefly demonstrate how differences in PC composition on oxDND and HPHT ND affect their toxicity.

#### 3.4.1 Hemolytic Activity of NDs with and without PC

Hemolysis assay is a standard method to evaluate the compatibility of NPs with RBC, as excessive hemolysis can lead to diseases such as anemia and jaundice.^35,36^ Therefore, assessing the hemolytic potential is an important step in determining the safety of NDs intended for biomedical uses.

The hemolytic activity of oxDND and HPHT ND was evaluated in the presence and absence of preformed PC (Figure 4 and Table 3). Across all tested conditions, the percentage of hemolysis remained low (Table 3), meeting the ASTM E2524–08 criterion for nanomaterials (<5%).^37^ Statistical analysis showed no significant differences in hemolytic activities between NDs with and without preformed PC (*p* > 0.05). This indicates that PC formation, whether HSA-dominant or a mix of HSA-APO A1, does not substantially alter the interaction of NDs with RBC. The results are consistent with previous reports indicating that NDs generally exhibit minimal hemolytic activity, which may be attributed to their low aspect ratio and strong protein binding capacity.^38,39^ Furthermore, the presence of PC, preformed or formed during incubation with RBC, reduces ND aggregation under physiological condition.^38^ Overall, both NDs, with or without preformed PC, exhibited hemocompatibility under the tested conditions.

**Figure 4:**
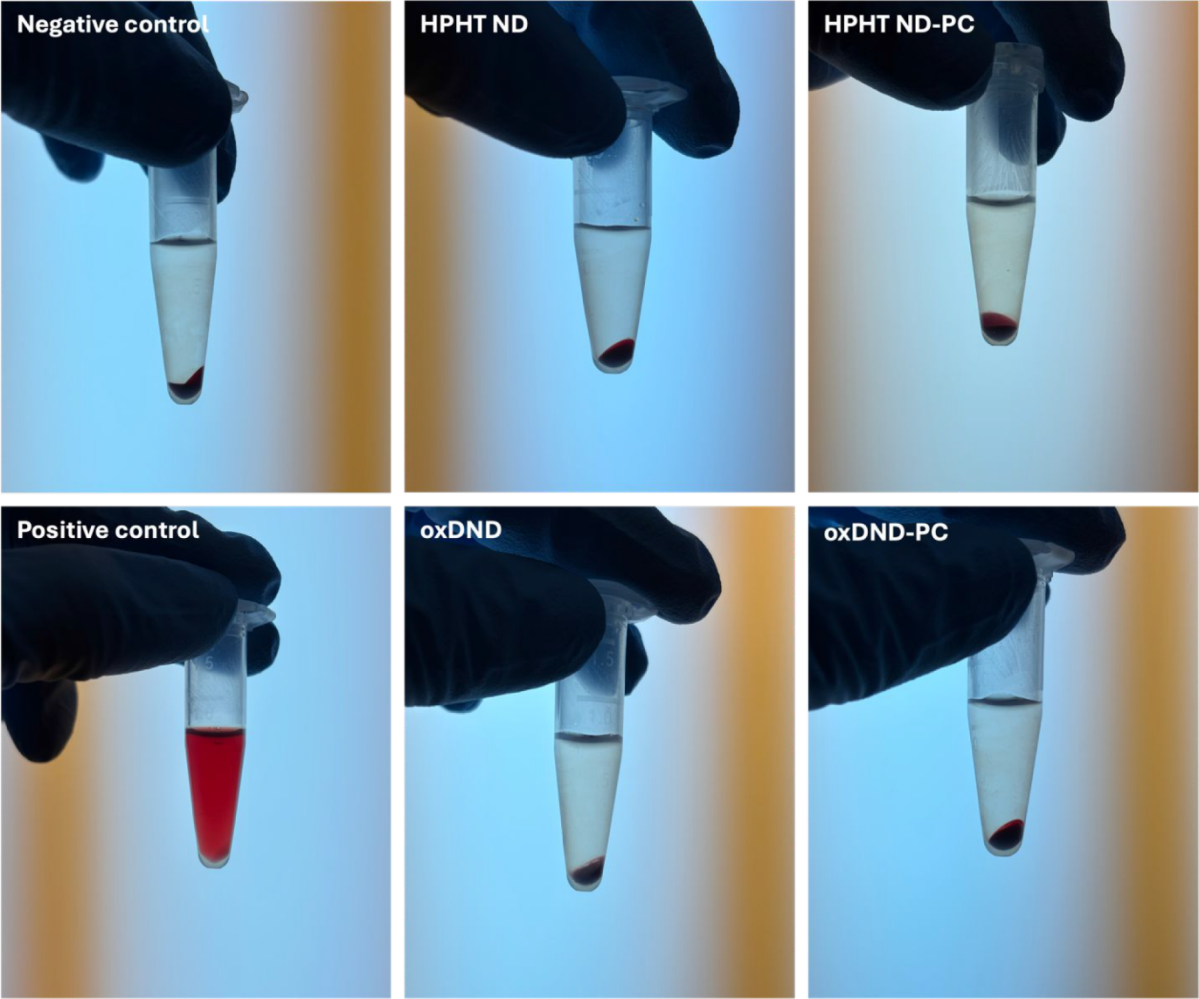
Hemolytic activity of oxDND and HPHT ND with or without preformed PC. All values were below <5%, with no significant differences observed (*p* > 0.05) as shown in Table 3.

**Table 3:** Percentage of Hemolysis Induced by oxDND and HPHT ND, with and without Preformed Protein Corona^a^

Sample	Hemolysis %
HPHT ND	0.40 ± 0.10
HPHT ND-PC	0.42 ± 0.07
oxDND	0.30 ± 0.10
oxDND-PC	0.32 ± 0.06
Positive control	100
Negative control	0

a Saline solution and Triton X-100 were used as negative and positive controls , respectively. Data represent mean values from triplicate measurements (*n* = 3). The calculated *p*-values for comparisons within the same type of ND were greater than 0.05, indicating that the observed differences in hemolytic activity were not statistically significant (n.s., *p* > 0.05).

#### 3.4.2 The Effects of NDs with Varying PC Compositions on A549 Cell Viability

Figure 5a shows the bright field images of A549 cells before and after their exposure to NDs with and without preformed PC. When exposed to 100 μg/mL of bare oxDND, a dramatic alteration in A549 cell morphology was observed. In contrast, cells exposed to oxDND with an HSA-dominant PC retained their morphology. Significant cell damage was also observed when cells were treated with bare HPHT ND. However, unlike oxDND, the presence of APO A1-HSA PC on HPHT ND did not reduce the damage to the cells. The viability of A549 cells exposed to NDs, with and without preformed PC, was quantitatively assessed using the CCK-8 assay. As shown in Figure 5b, the viability of A549 cells decreased by approximately 30% when exposed to oxDND without preformed PC. Consistent with the bright field images, cell viability improved when exposed to oxDND with an HSA-dominant PC (with a drop of only ∼20%). For cells exposed to bare HPHT ND, the viability also dropped by approximately 30%. Surprisingly, it further decreased to ∼50% when A549 cells were exposed to HPHT ND with an APO A1-HSA corona. The altered cell morphology and cell survival rate indicate that the HPHT ND with APO A1-HSA corona has a higher cytotoxicity than the oxDND with HSA corona.

**Figure 5:**
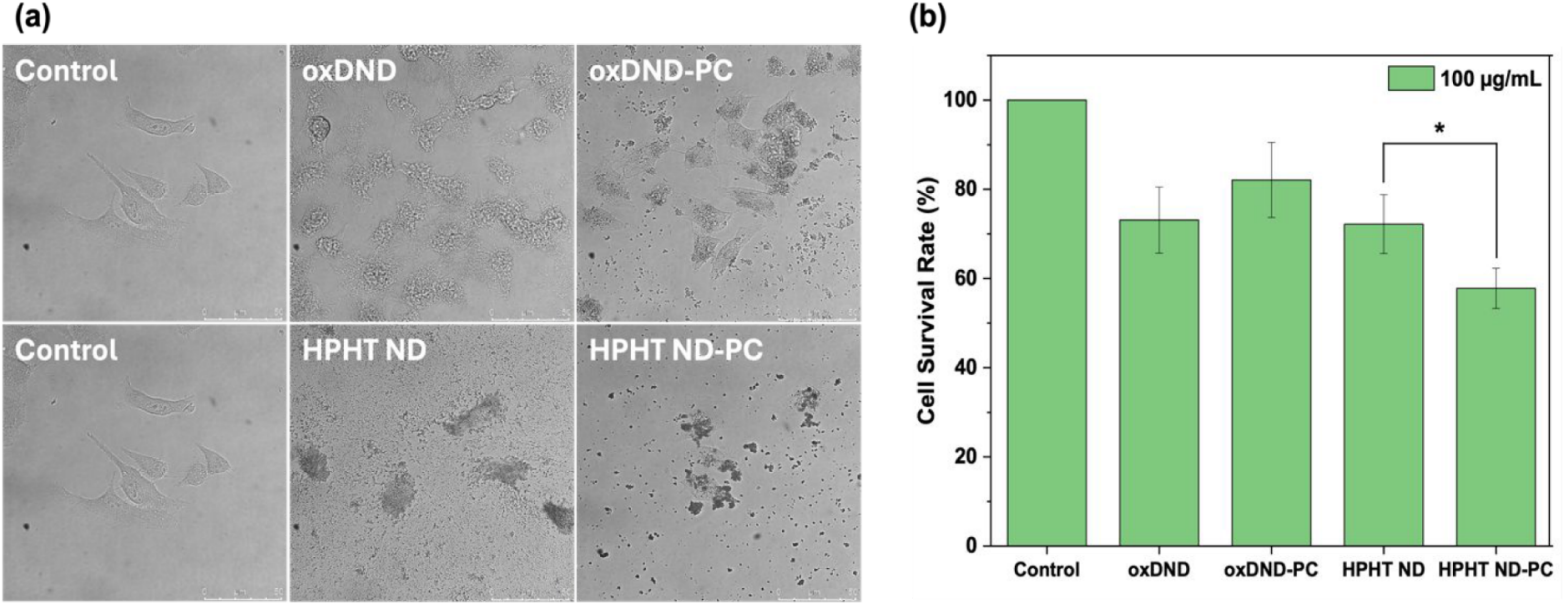
PC influences the cytotoxicity of NDs. (a) Bright field images of A549 cells before and after exposure to oxDND and HPHT ND, with and without PC. (b) Survival rate of A549 cells before and after exposure to NDs, with and without PC, measured using the CCK-8 assay (*n* = 3; *p < 0.05).

Since HSA constitutes 60% of the total serum proteins, numerous studies focused on understanding the formation of HSA corona on NPs and its impact on biological toxicity.^4,7,15,40–43^ For instance, Khanal et al. demonstrated that pristine NDs coated with a BSA corona exhibit less toxicity toward Fao cells than pristine NDs with a fibronectin corona.^15^ The reduced cytotoxicity was associated with the level of ND’s internalization, which was qualitatively lower for NDs with the BSA corona. The authors further noted that serum proteins, such as albumins, contain essential nutrients for cell growth and viability. Hence, when BSA binds to the surface of NDs, their internalization facilitates the direct delivery of these nutrients to the cells. However, it must be acknowledged that this may not be applicable to all types of proteins and NPs. Khanal et al.’s observations are consistent with earlier studies about the BSA corona-mediated mitigation of GO’s cytotoxicity.^7,41^ Using both experimental and theoretical methods, Duan et al. revealed that BSA-corona reduced the cellular uptake of GO, hence reducing its cytotoxicity.^41^ Their simulation validated that the bulkier surface caused by the BSA corona hindered the direct insertion of GO into the cell membrane.

It is important to acknowledge that one limitation of our study is the current lack of understanding of APO A1’s role in modulating the cytotoxic effects of NPs. Only a few studies have examined APO A1 coronas, with some showing that they can reduce NP uptake and lower cytotoxic and inflammatory effects.^44–47^ The potential role of APO A1 in treating lung diseases such as lung cancer and asthma, through its antitumor, anti-inflammatory, antioxidant, and immune-modulating effects,^48–54^ is also worthy to consider when interpreting the reduction in A549 cell viability. These properties have also been applied in the design of APO A1-based NPs for drug delivery and immune modulation,^55,56^ although such applications are not specific to lung diseases. While the literature offers useful insights into the functions of APO A1, further targeted studies are needed to determine how APO A1-enriched HPHT NDs affect lung epithelial cell viability. Our findings should be interpreted as a preliminary step in this complex field.

## 4 Conclusions

MALDI-TOF MS proved to be a valuable tool for identifying the high-abundance human serum proteins adsorbed on oxDND and HPHT ND, providing insights into the evolution and composition of their corona. At a serum protein concentration of 5.3 μg/mL, both NDs are primarily coated with apolipoprotein A1 (APO A1; Mw = 28,000 Da) As serum protein concentration decreased to 1.6 μg/mL, oxDND becomes dominated by human serum albumin (HSA; Mw = 66,500 Da), whereas HPHT ND exhibited a mixed APO A1–HSA corona. Structural differences between the two NDs also influenced protein adsorption. The oxDND with a larger pore diameter (9.41 nm) provides more access to proteins with larger Mw, like HSA, while the smaller pore diameter (3.39 nm) of HPHT preferentially diffuses low Mw proteins. Beyond pore size, differences in surface chemistry and carbon structure indicated by Raman and XPS analyses may also contribute to the observed corona profiles. These observations emphasize the complex nature of corona formation, influenced by protein abundance, affinity, and kinetics, as well as ND’s properties.

Hemolysis assays confirmed that both NDs exhibited good hemocompatibility, as neither with nor without preformed corona NDs induced significant RBC lysis. In contrast, microscopy and cell viability assays demonstrate that differences in corona composition affect the cytotoxicity of oxDND and HPHT ND toward A549 cells. While oxDND with an HSA-dominant corona shows slightly reduced toxicity, HPHT with an APO A1-HSA corona exhibits higher cytotoxicity. These results highlight that proteins composing the corona modulate ND cytotoxicity in a system-dependent manner, with implications for drug delivery and nanomedicine applications. Beyond these applications, NDs exhibiting preferential adsorption of highly abundant plasma proteins, such as albumins, apolipoproteins, and immunoglobulins, may also serve as useful tools in protein depletion strategies. By selectively binding these dominant proteins, NDs could facilitate the enrichment and subsequent detection of low-abundance biomolecules, which is particularly beneficial in applications such as proteomic analysis, biomarker discovery, and early disease diagnostics.^57–61^

## Supplementary Material



## Notes

Previously published ND characterization; time-dependent protein corona stability; FTIR analysis; consecutive ND-protein extractions; mass spectrometry analysis of serum proteins; Bradford assay quantification; hydrodynamic size and zeta potential measurements; Mascot protein identification (PDF)
