# Supplementary material for: Profiling High-Abundance Serum Proteins in the Corona of Nanodiamonds Using Mass Spectrometry
Source: Langmuir. 2026 Mar 16;42(14):9887–97. doi: 10.1021/acs.langmuir.5c06674 (PMC13085801; doi:10.1021/acs.langmuir.5c06674)
Supplement: Supplementary file 1 [file la5c06674_si_001.pdf]

Supporting Information

Profiling High-Abundance Serum Proteins in the Corona of Nanodiamonds Using Mass Spectrometry

Mhikee Janella N. Descanzo[1], Yu-Chung Chen[1], Ming-Chi Chung[2], Nguyen Nghiem Bich Ngoc[2], Avinash A. Patil[1], Po-Chi Soo[4], Yu-Tze Horng[4], Chia-Liang Cheng[1], Huan-Cheng Chang[3], Ruey-Yi Chang[2], and Wen-Ping Peng[1,*]

[1]*Department of Physics, National Dong Hwa University, Shoufeng, Hualien 97401, Taiwan*

[2]*Department of Biochemical and Molecular Medical Sciences, National Dong Hwa University, Hualien 97401, Taiwan*

[3]*Institute of Atomic and Molecular Science, Academia Sinica, Taipei 10617, Taiwan*

[4]*Department of Laboratory Medicine and Biotechnology, Tzu Chi University, Hualien 97004, Taiwan*

To whom correspondence should be addressed, e-mail: pengw@gms.ndhu.edu.tw

Prepared for *Langmuir* (Article)

Contract/grant sponsor: NSTC 114-2112-M-259-009-, 114-2923-M-259-001-MY3, 114-2112-M-259-003-, and 113-2112-M-259-002- (W.P.P.)

The supporting information contains:

18 Pages, 4 Sections, 6 Figures, and 2 Tables.

Content Section

Section 1. Previously Published ND Characterization Results S-4

Section 2. Time-Dependent Stability of Protein Corona (PC) S-5

Section 3. Fourier-Transform Infrared (FTIR) Spectroscopy S-6

Section 4. Consecutive ND-Protein Extraction S-6

Figures

Figure S1. Time-dependent evolution of protein corona on the surfaces of a) oxDND and b) HPHT ND. S-7

Figure S2. Mass spectra of 16 µg/mL total serum proteins extracted with 100 µg/mL oxDND and HPHT-ND. These conditions, used in cell survival experiments, facilitate microscopic visualization and are comparable to lower concentrations of serum proteins (1.6 µg/mL) and NDs (10 µg/mL). S-8

Figure S3. Validation of the functional groups present on the surface of HPHT ND and oxDND using FTIR spectroscopy. S-9

Figure S4. **Consecutive ND-protein extractions**: blue mass spectra represent the serum proteins adsorbed to the surface of **oxDND** during the first extraction experiment at total protein concentration of a) 5.3, b) 2.7, c) 1.6, and d) 1.1 µg/mL. The red mass spectra represent the remaining proteins in the supernatant observed during the second and third extractions. The asterisk (*) indicates the singly and doubly charged states of APO A1. .... S-10

Figure S5. **Consecutive ND-protein extractions**: blue mass spectra represent the serum proteins adsorbed to the surface of **HPHT ND** during the first extraction experiment at total

protein concentration of a) 5.3, b) 2.7, c) 1.6, and d) 1.1 μg/mL. The red mass spectra represent the remaining proteins in the supernatant observed during the second and third extractions. The asterisk (*) indicates the singly and doubly charged states of APO A1. …………………. S-11

Figure S6. **Bradford assay**: (a) Standard calibration curve obtained using bovine serum albumin (BSA) in the concentration range of 0 – 50 μg/mL. Quantification of total amount of proteins adsorbed on (b) oxDND and (c) HPHT ND after exposure to varying concentrations of human serum (2.7 – 13 μg/mL). ……………………………………………………….. S-12

Tables

Table S1. Measured hydrodynamic size (*z-average*) and zeta potential of oxDND and HPHT ND before and after incubation with serum at different concentrations. …………………. S-13

Table S2. MASCOT analysis of serum proteins extracted using oxDND. ………………. S-14

References ……………………………………………………………………………... S18

Section 1. Previously Published NDs Characterization Results

Our research group has previously performed detailed physicochemical characterization of the two nanodiamond (ND) types used in this study: oxidized detonation NDs (oxDND) and high-pressure high-temperature NDs (HPHT NDs). A brief summary of the relevant findings is provided below[1].

Raman spectroscopy showed that both NDs possess a crystalline diamond core, evidenced by the peak near ~1331 cm^{-1} and by weaker D/G bands associated with sp^2 carbon. Notably, oxDND displayed a comparatively stronger sp^2 contribution, consistent with its detonation-derived surface structure, whereas HPHT NDs exhibited a cleaner diamond signature.

X-ray photoelectron spectroscopy (XPS) analysis further differentiated the two ND types. The oxDND exhibited a higher abundance of oxygen-containing functional groups (C–O, C=O), indicating extensive surface oxidation, while HPHT NDs showed a more carbon-rich C 1s profile with lower oxygen content. These differences highlight the more hydrophilic, highly oxidized surface of oxDND relative to the less oxidized HPHT ND surface.

These prior characterizations confirm that oxDND and HPHT NDs differ significantly in both surface chemistry and sp^2/sp^3 carbon distribution. These are some factors that are expected to influence their protein adsorption behavior and help contextualize the protein corona (PC) compositions observed in the present study.

Section 2. Time-Dependent Stability of Protein Corona (PC)

PC formation is typically classified into two types: **hard corona** and **soft corona**. The hard corona consists of proteins that rapidly bind (within seconds to minutes) and tightly bound to the NP surface. In contrast, the soft corona comprises loosely bound, low-affinity proteins that are continuously exchanged depending on their abundance and binding affinity[2-4]. A time-dependent experiment was conducted to assess the stability of the corona composition, helping to standardize the method used in this study. Proteins attached to the ND surface were analyzed after incubation with human serum for 5-, 30-, and 180-min. **Figure S1a** shows that, regardless of the incubation time, the oxDND is mainly covered by a single protein with a molecular weight (Mw) of 66,500. Likewise, variations in incubation time did not affect the proteins attached to the surface of HPHT ND, which have Mw ranging from 9,000 to 15,000, as shown in **Figure S1b**. The persistence of these protein profiles over time confirms that the proteins detected by MS represent the hard corona. Therefore, a standard 30 min-incubation was chosen for all subsequent experiments in this study.

Section 3. Fourier-Transform Infrared (FTIR) Spectroscopy

The functional groups on the surface of oxDND and HPHT ND were measured using an MB154 FTIR spectrometer interferometer (ABB Inc, Bomem, Zurich, Switzerland) with the sample in ambient environment. Prior to the measurement, 2 mg of ND powders were separately dissolved in 60 μL of DDW. Then, 20 μL of each solution was carefully dropped on a silicon wafer and air-dried to form a thin film. Results are presented in **Figure S2**.

Section 4. Consecutive ND-Protein Extraction

The supernatants were analyzed for protein content by performing **consecutive ND-protein extractions** until no signal was detected. If no protein signal was observed during the second extraction, it was assumed that the ND particles had adsorbed all the proteins during the first extraction. Conversely, if ion signals were still detected during the second extraction, it was assumed that the NDs had reached their maximum adsorption capacity for proteins. Multiple extractions were conducted based on the adsorption capacity of each ND type and the total protein concentration. Results for oxDND and HPHT are demonstrated in **Figures S4** and **S5**, respectively.

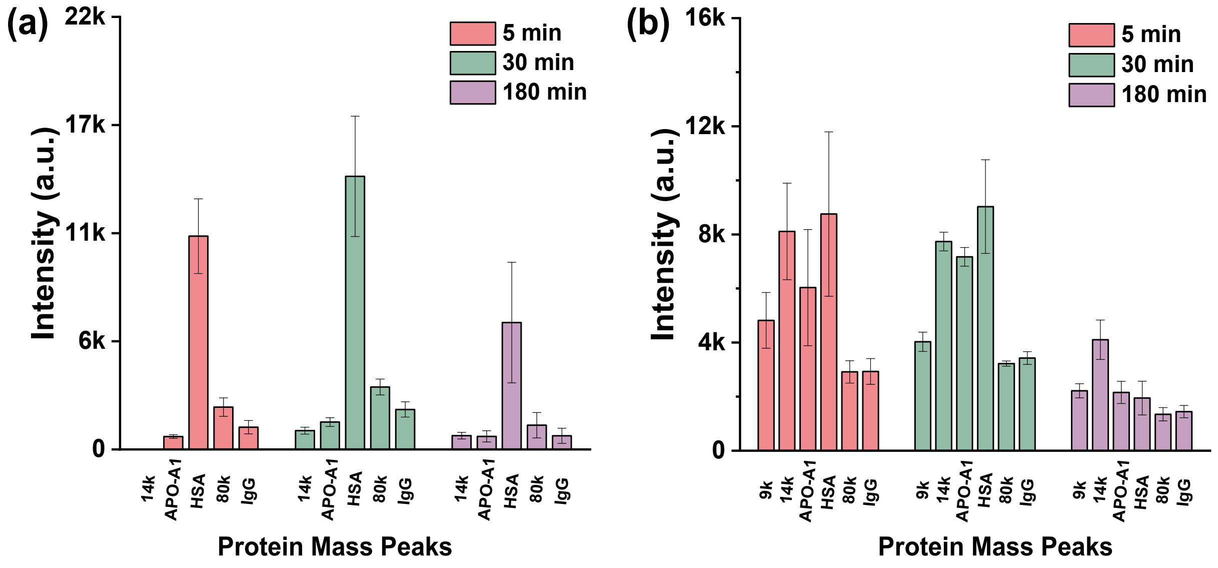

Figure S1. Time-dependent evolution of protein corona on the surfaces of a) oxDND and b) HPHT ND.

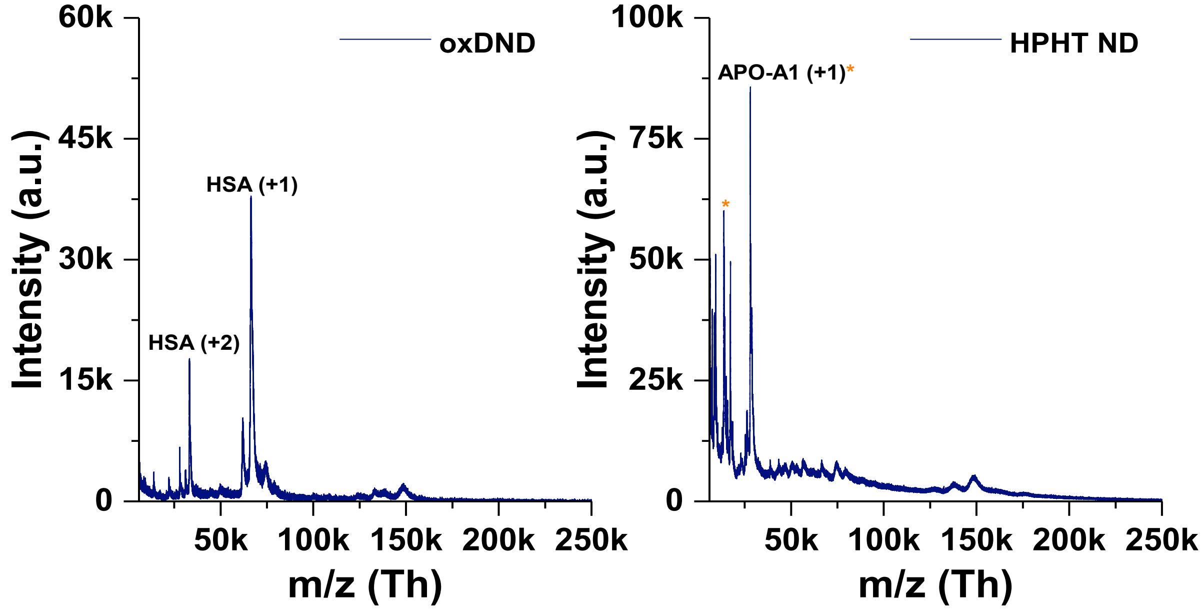

Figure S2. Mass spectra of 16 µg/mL total serum proteins extracted with 100 µg/mL oxDND and HPHT-ND. These conditions, used in cell survival experiments, facilitate microscopic visualization and are comparable to lower concentrations of serum proteins (1.6 µg/mL) and NDs (10 µg/mL).

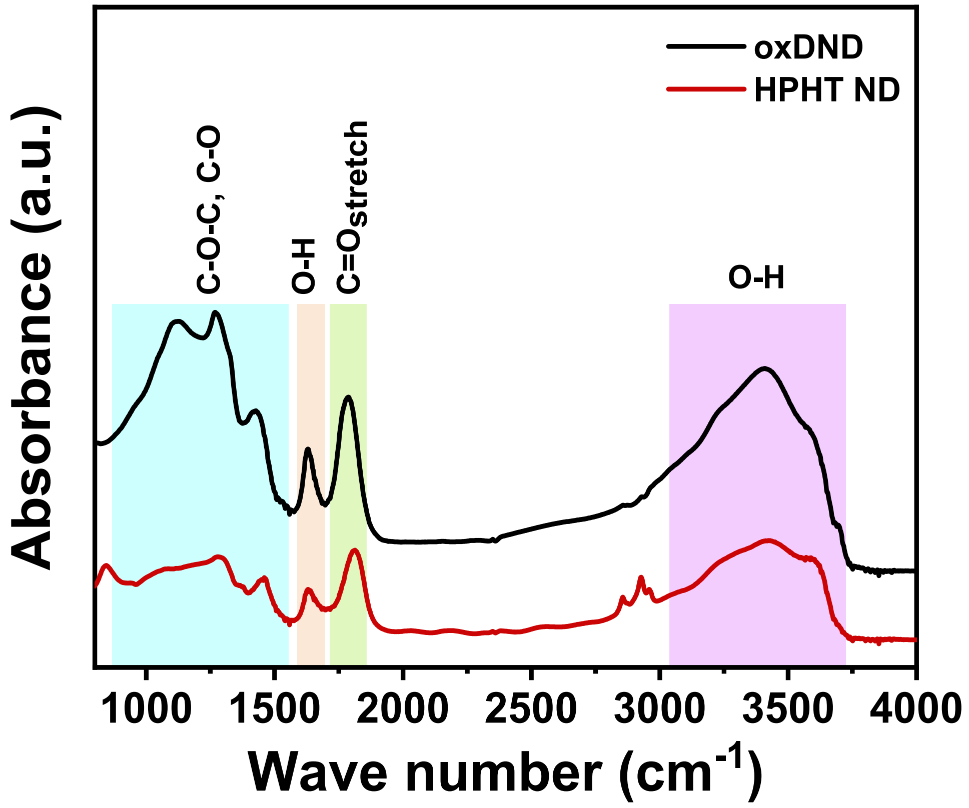

Figure S3. Validation of the functional groups present on the surface of HPHT ND and oxDND using FTIR spectroscopy.

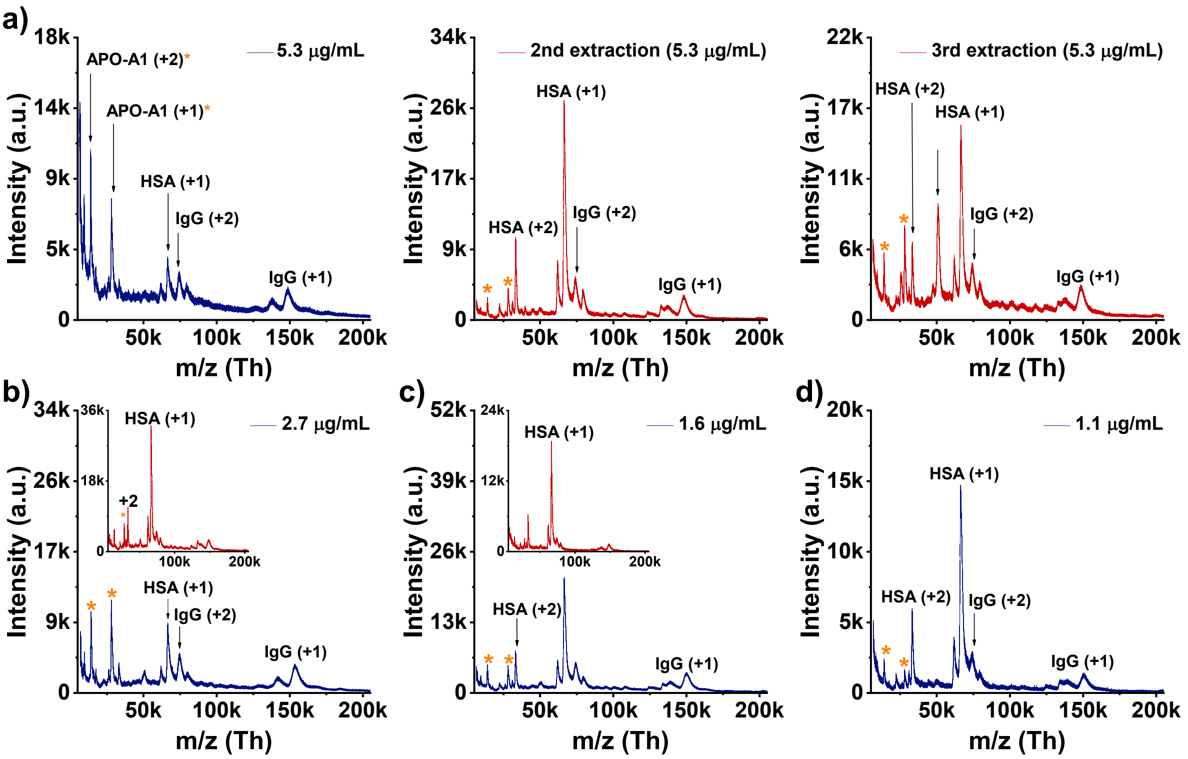

Figure S4. **Consecutive ND-protein extractions**: blue mass spectra represent the serum proteins adsorbed to the surface of **oxDND** during the first extraction experiment at total protein concentration of a) 5.3, b) 2.7, c) 1.6, and d) 1.1 µg/mL. The red mass spectra represent the remaining proteins in the supernatant observed during the second and third extractions. The asterisk (*) indicates the singly and doubly charged states of APO A1.

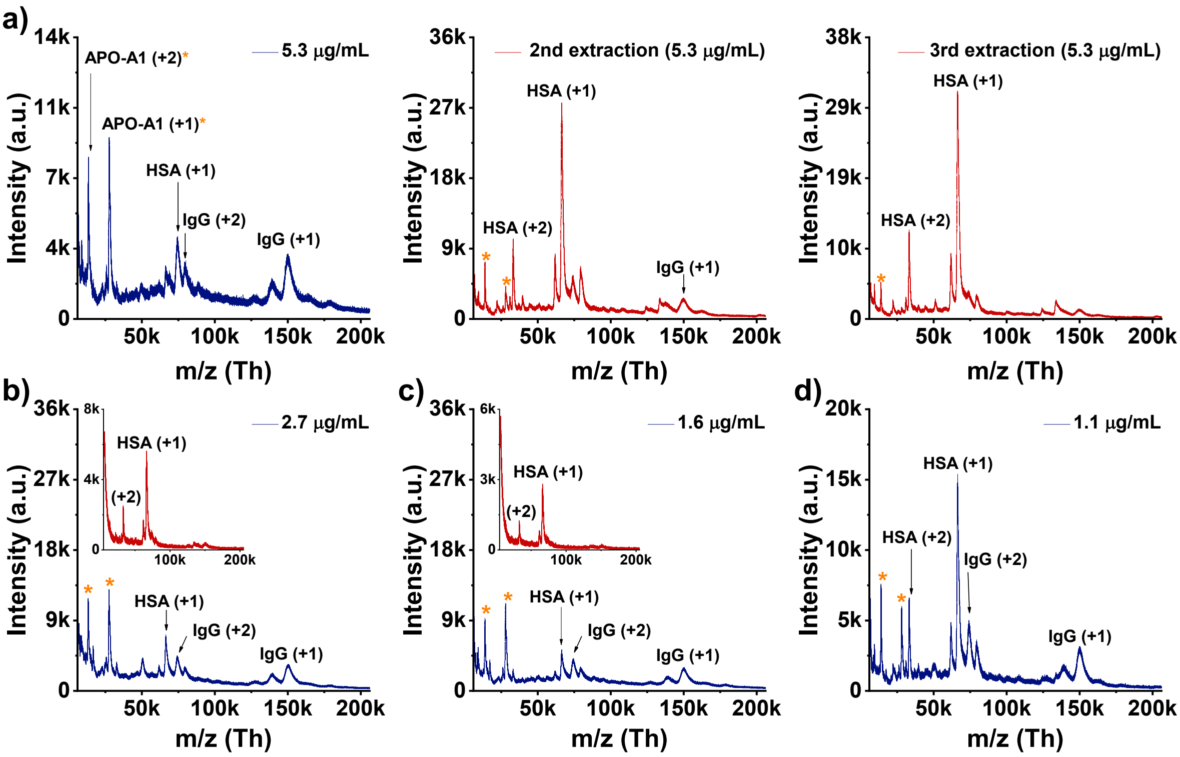

Figure S5. **Consecutive ND-protein extractions**: blue mass spectra represent the serum proteins adsorbed to the surface of **HPHT ND** during the first extraction experiment at total protein concentration of a) 5.3, b) 2.7, c) 1.6, and d) 1.1 µg/mL. The red mass spectra represent the remaining proteins in the supernatant observed during the second and third extractions. The asterisk (*) indicates the singly and doubly charged states of APO A1.

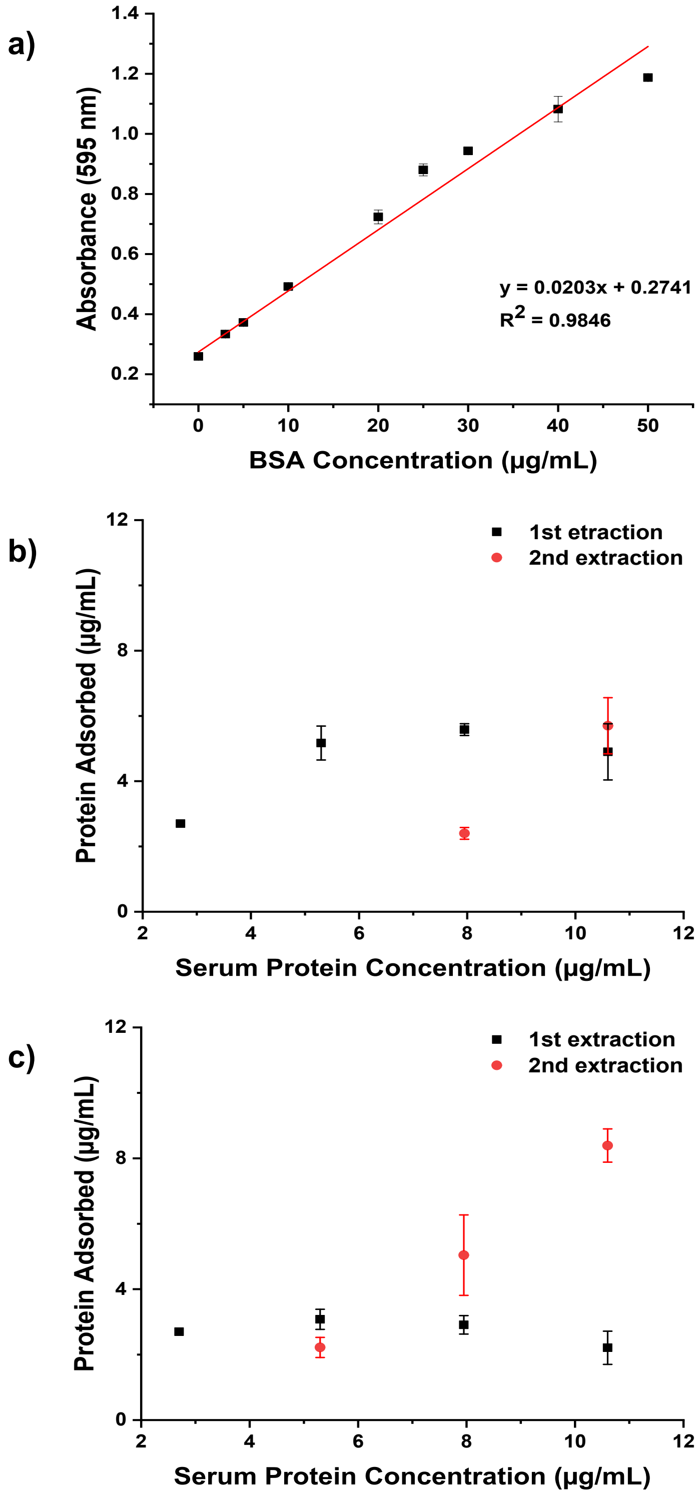

Figure S6. **Bradford assay**: (a) Standard calibration curve obtained using bovine serum albumin (BSA) in the concentration range of 0 – 50 µg/mL. Quantification of total amount of proteins adsorbed on (b) oxDND and (c) HPHT ND after exposure to varying concentrations of human serum (2.7 – 13 µg/mL).

Table S1. Measured hydrodynamic size (*z-average*) and zeta potential of oxDND and HPHT ND before and after incubation with serum at different concentrations.

Sample	Total Serum Protein Concentration (µg/mL)	Hydrodynamic Diameter (nm)	ζ-Potential (mV)
oxDND	0	144 ± 2	-49 ± 3
	5.3	239 ± 3	-20 ± 0.7
	1.6	196 ± 3	-26 ± 2
	1.1	191 ± 2	-39 ± 0.8
HPHT ND	0	158 ± 2	-49 ± 0.9
	5.3	188 ± 3	-17 ± 0.8
	1.6	174 ± 3	-27 ± 0.5
	1.1	173 ± 2	-29 ± 1

Table S2. MASCOT analysis of serum proteins extracted using oxDND.

(a) **HSA sequence coverage: 54%**

Only matched peptides (shown in **bold red**) were listed below.

FLFSSAYSR**G**	**VFRR**DAHKSE	VAHR**FKDLGE**	**ENFKALVLIA**
**FAQYLQQCPF**	**EDHVKLVNEV**	**TEFAK**TCVAD	VATLR**ETYGE**
**MADCCAKQEP**	**ERNECFLQHK**	**DDNPNLPQLV**	**RPEVDVMCTA**
**FHDNEETFLK**	FAKRY**AAFT**	**ECCQAADKAA**	**CLLPK**LDELR
GERAFK**AWAV**	**ARLSQRFPKA**	**EFAEVSK**LVT	DLTK**VHTECC**
**HGDDLLECADD**	**R**ADLAK**YICE**	**NQDSISSK**LK	**SHCIAEVEND**
**EMPADLPSLA**	**ADFVESKDVC**	**K**NYAEAKDVF	R**HPDYSVVLL**
**LRLAK**TYETT	LIK**QNCELFE**	**QLGEYKFQNA**	**LLVRYTKKVP**
**QVSTPTLVEV**	**SR**NLGKVGSK	CCKHPEAK**RM**	**PCAEDTLSVV**
**LNQLCVLHEK**	CTESLVNR**RP**	**CFSALEVDET**	**YVPKEFNAET**
**FTFHADICTL**	**SEKER**QIKKQ	TALVELVK**HK**	**PK**ATKEQLK**A**
**VMDDFAAFVE**	**K**CCKADDKET		

Start-End	Observed	Mr(expt)	Missed	Peptide
20 – 24	634.7650	633.7577	1	R.GVFRR.D
35 – 44	1226.6690	1225.6617	1	R.FKDLGEENKK.A
37 – 65	3424.7100	3423.7027	1	K.DLGEENFKALVLIAFAQYLQQCPFEDHVK.L
66 – 75	1149.6500	1148.6427	0	K.LVNEVTEFAK.T
106 – 117	1434.5730	1433.5657	0	R.ETYGEMADCCAK.Q

118 – 122	657.8820	656.8747	0	K..QEPER.N
123 - 138	1996.6540	1995.6467	1	R.NECFLQHKDDNPNLPR.L
131 – 138	940.3790	939.4410	0	K.DDNPNLPR.L
139 – 160	2667.8540	2665.2516	0	R.LVRPEVDVMCTAFHDNEETFL K.K + Oxidation (M)
187 – 205	2124.1210	2123.9802	1	K.AAFTECCQAADKAACLLPK.L
237 – 246	1157.5480	1156.6465	1	K.AWAVARLSQR.F
247 – 257	1252.7730	1251.6499	1	R.FPKAEFAEVSK.L
265 – 281	2085.9970	2085.8303	0	K.VHTECCHGDLLECADDR.A
287 – 298	1443.7500	1442.6347	0	K.YICENQDSISSK.L
311 – 337	2990.0450	2989.3321	0	K.SHCIAEVENDEMPADLPSLAAD DFVESK.D + Oxidation (M)
338 – 341	521.5700	520.2315	0	K.DVCK.N
362 - 375	1624.6590	1622.9508	1	R.HPDYSVVLLLRLAK.T
414 – 434	2598.3460	2598.2900	1	K.QNCELFEQLGEYFKQNALLVR.Y
427 – 434	960.5030	959.5552	0	K.FQNALLVR.Y
435 - 438	539.7490	538.3115	1	R.YTKK.V
439 – 452	1511.8520	1510.8355	0	K.VPQVSTPTLVEVSR.N
469 – 490	2690.7320	2698.3026	1	K.RMPCAEDYLSVVLNQLCVHE K.T + Oxidation (M)
509 – 524	1911.0660	1909.9244	0	R.RPCFSALEVDETYVPK.E
525 – 545	2543.7060	2544.1591	1	K.EFNAETFTFHADICTLSEKER.Q
559 – 562	509.7240	508.3121	0	K.HKPK.A
570 - 581	1342.6690	1341.6257	0	K.AVMDDFAAFVEK.C

(b) **APOA1 sequence coverage: 71%**

Only matched peptides (shown in **bold red**) were listed below.

LFLTGSQAR**H**	**FWQQDEPPQS**	**PWDRV**KDLAT	VYVDVLK**DSG**
**RDYVSQFEGS**	**ALGKQLNKL**	**LDNWDSVTST**	**FSK**LREQLGP
**VQPYLDDFQK**	**KWQEEMELYR**	QK**VEPLRAEL**	**QEGAR**QK**LHE**
**LQEKLSPLGE**	**EMRDRARAHV**	**DALRTHLAPY**	**SDELR**QRLAA
R**LEALKENGG**	**ARLAEYHAKA**	**TEHLSTLSEK**	**AKPALEDLRQ**
**GLLPVLESFK**	**VSFLSALEEY**	**TKKLNTQ**	

Start-End	Observed	Mr(expt)	Missed	Peptide
20 – 36	2179.7390	2178.7317	1	R.HFWQQDEPPQSPWDRVK.D
48 – 64	1815.4220	1814.4147	1	K.DSGRDYVSQFEGSALGK.Q
65 – 83	2208.4960	2207.4887	1	K.QLNKLLDNWDSVTSTFSK.L
70 – 83	1612.8690	1611.8617	0	K.LLDNWDSVTSTFSK.L
121 – 130	1252.7920	1251.7847	0	K.VQPYLDDFQK.K
121 – 131	1380.7310	1379.7237	1	K.VQPYLDDFQKK.W
131 – 140	1427.6900	1426.6827	1	K.KWQEEMELYR.Q + Oxidation (M)
132 – 140	1299.7100	1298.7027	0	K.WQEEMELYR.Q + Oxidation (M)
143 – 155	1467.7960	1466.7887	1	K.VEPLRAELQEGAR.Q
158 – 173	1910.1310	1909.1237	1	K.LHELQEKLSPLGEEMR.D
165 – 173	1047.5620	1046.5547	0	K.LSPLGEEMR.D + Oxidation (M)
165 - 175	1318.7600	1317.7527	1	K.LSPLGEEMRDR.A + Oxidation (M)
174 – 177	517.4100	516.4027	1	R.DRAR.A

178 – 184	781.1160	780.1087	0	R.AHVDALR.T
178 – 195	2062.8400	2061.8327	1	R.AHVDALRTHLAPYSDELR.Q
185 – 195	1301.7570	1300.7497	0	R.THLAPYSDELR.Q
202 – 212	1157.7500	1156.7427	1	R.LEALKENGGAR.L
213 – 219	831.1230	830.1157	0	R.LAEYHAK.A
213 – 230	2026.8530	2025.9457	1	R.LAEYHAKATEHLSTLSEK.A
220 – 230	1215.7970	1214.7897	0	K.ATEHLSTLSEK.A
231 – 239	1012.6020	1011.5947	0	K.AKPALEDLR.Q
240 – 262	2598.1050	2597.0977	1	R.QGLLPVLESFKVSFLSALEEYTK.K
251 – 260	1386.7730	1385.7657	0	K.VSFLSALEEYTK.K
251 – 263	1514.6360	1513.6287	1	K.VSFLSALEEYTKK.L
263 – 267	603.6860	602.6787	1	K.KLNTQ.-

References

(1) Patil, A. A.; Descanzo, M. J. N.; Agcaoili, J. B. A.; Chiang, C.-K.; Cheng, C.-L.; Chang, H.-C.; Peng, W.-P. Carboxylated/Oxidized Diamond Nanoparticles for Quantifying Immunoglobulin G Antibodies Using Mass Spectrometry. *ACS Applied Nano Materials* **2021**, *4* (9), 8922-8936. DOI: 10.1021/acsanm.1c01553.

(2) Aggarwal, P.; Hall, J. B.; McLeland, C. B.; Dobrovolskaia, M. A.; McNeil, S. E. Nanoparticle interaction with plasma proteins as it relates to particle biodistribution, biocompatibility and therapeutic efficacy. *Advanced Drug Delivery Reviews* **2009**, *61* (6), 428-437. DOI: https://doi.org/10.1016/j.addr.2009.03.009.

(3) García-Álvarez, R.; Vallet-Regí, M. Hard and Soft Protein Corona of Nanomaterials: Analysis and Relevance. In *Nanomaterials*, 2021; Vol. 11.

(4) Weiss, A. C. G.; Krüger, K.; Besford, Q. A.; Schlenk, M.; Kempe, K.; Förster, S.; Caruso, F. In Situ Characterization of Protein Corona Formation on Silica Microparticles Using Confocal Laser Scanning Microscopy Combined with Microfluidics. *ACS Applied Materials & Interfaces* **2019**, *11* (2), 2459-2469. DOI: 10.1021/acsami.8b14307.
